# Prevalence and Pattern of Ocular Trauma in a Secondary Eye Care Center in Nepal: A Descriptive Cross-sectional Study

**DOI:** 10.31729/jnma.8599

**Published:** 2024-06-30

**Authors:** Hom Bahadur Gurung, Kalpana Singh, Mohini Shrestha, Aleena Gauchan, Adita Dhoju, Sunil Thakali

**Affiliations:** 1 Department of Oculoplasty, Tilganga Institute of Ophthalmology, Gaushala, Kathmandu, Nepal; 2 Department of Glaucoma, Tilganga Institute of Opnthalmology, Gaushala, Kathmandu, Nepal; 3 Department of Ophthalmology, Hetauda Community Eye Hospital, Hetauda, Nepal; 4 Tilganga Institute of Ophthalmology, Gaushala, Kathmandu, Nepal

**Keywords:** *accidents*, *agriculture*, *eye injury*, *prevalence*, *welding*

## Abstract

**Introduction:**

Ocular trauma stands as the leading preventable cause of monocular blindness worldwide. The aim of the study was to calculate the prevalence of ocular trauma and the circumstances, causes, and types of ocular injuries at emergency department of Community Eye Hospital.

**Methods:**

This was a descriptive cross-sectional study conducted retrospectively on patients presenting to the Emergency Department in the year 2020. Ethical clearance was received from the Institutional Review Board with reference number 12/2021. Data collection commenced in April 2021, following the approval in March 2021. Descriptive statistics with mean and frequency were used for analysis.

**Results:**

Among the 6526 emergency cases visiting the emergency department of Hetauda community eye hospital the prevalence of ocular trauma was 2143 (32.83%; 95% CI: 31.69%-33.97%). The mean age among the 2143 trauma cases was 33.55±15.63 years. Among them, 1851 (86.40%) fell in the working age group. The male-to-female ratio was 3:1. Occupational injuries due to welding, agriculture and industries were in 604 (28.19%) of all ocular injuries.

**Conclusions:**

The prevalence of ocular trauma in our study was higher than other studies. Occupational ocular trauma mainly welding injury, cement factory injury and agricultural injury are common cause of ocular trauma.

## INTRODUCTION

Ocular trauma, a part of ocular emergency is a major public health problem and a leading cause of preventable monocular blindness worldwide.^1^ The gravity of ocular trauma may range from mild discomfort to complete loss of vision.

The pattern and implications of ocular trauma differ by region. Understanding the prevalence, reasons, timing and location of trauma is important for devising appropriate prevention strategies targeted at vulnerable population and places.^2^ Stringent laws in developed countries have decreased the prevalence of trauma, while it is not same for developing countries.^3,4^

The aim of the study was to find out the prevalence of ocular trauma in our hospital and identify the pattern of ocular trauma in the region so as to allocate the limited resources toward preventing ocular morbidity and blindness.

## METHODS

This retrospective descriptive cross-sectional study was conducted in the patients presenting to the emergency department (ED) of Hetauda Community Eye Hospital (HCEH) in the year 2020. The study population included all patients visiting the hospital emergency department in the study period. Ethical clearance was received from the Institutional review board of Tilganga Institute of Ophthalmology with reference number 12/2021 on 19^th^ march 2021 and data collection was started from April 2021. The study is part of the ocular emergency study.^5^

Any form of injury in and around the eye was taken as ocular injury. All cases with a history of eye injury and /or diagnosed as ocular injury were included in the study. Data were retrieved from the hospital electronic record system and all patients attending the emergency department were searched for evidence of trauma. Ocular Trauma was classified as per the Birmingham Eye Trauma Terminology (BETT) classification.^6^

The number of follow-up visits and visual acuity at both presentation and the last follow-up were recorded. The circumstances of trauma were categorized as follows: accidental, domestic violence, leisure, physical assault, road traffic accidents, sports, welding /metal work and work place. If patient did not know the circumstance of injury it was kept as accidental.

Foreign bodies in conjunctiva and cornea were classified whenever clear identification was mentioned in data like iron, animal or plant products, wherever it was not clear it was grouped into foreign body conjunctiva. Data cleaning was performed using Microsoft Excel. Nominal, ordinal, and scale variables were expressed as percentages, frequencies, and appropriate measures of central tendency (mean, median, and range).

## RESULTS

**Figure 1:**
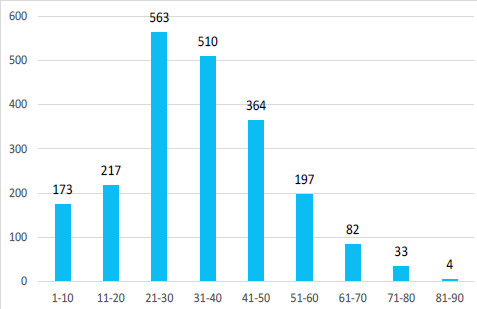
Age distribution of ocular trauma patients visiting ED of HCEH in 2020.

Among 6526 patients who visited the emergency department of Hetauda community Eye Hospital the prevalence of ocular trauma was 2143 (32.83%; 95% CI: 31.69%-33.97%). Out of these eye injury cases, 525 (24.50%) were females and 1618 (75.50%) were males. The mean age was 33.55±15.63 years and ranged from 1 to 87 years (Figure 1).

Out of the total injuries, 1484 (69.24%) occured in unclear or unknown circumstances. Other eye injuries were related to work 604 (28.19%), such as agricultural or industrial and welding or metal work. In work related injuries, welding and metal work injuries made up 379 (17.70%) cases and other workplace injuries constituted 225 (10.50%) cases. The injuries related to physical assult and sports or road traffic accident accounted for 28 (1.31%) each.

**Figure 2:**
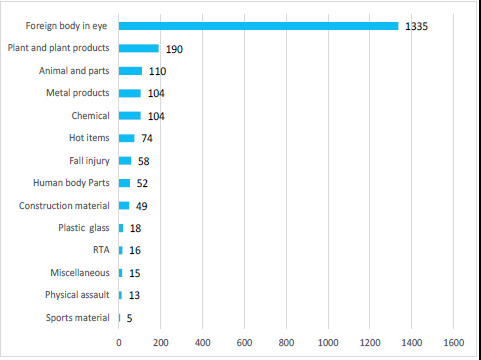
Bar chart showing number of cases by cause of trauma.

We identified 115 different reasons for these eye injuries. We grouped these causes into similar categories (Figure 2).

**Figure 3:**
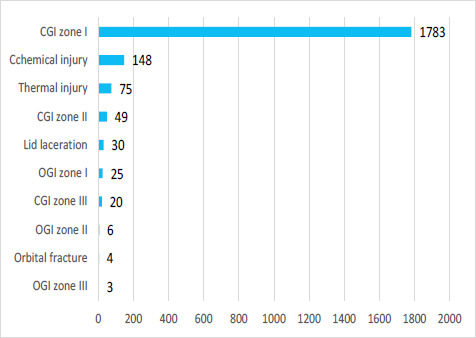
Bar chart showing number of cases by trauma classification.

Among the types of eye injuries, closed globe injuries in zone I (CGI zone I) were 1783 (83.20%), chemical injuries were 148(6.91%) and thermal injuries were 75(3.50%), (Figure 3). Chemicals of unknown origin, cement, superglue, vicks inhalers, paint, sprays, and insecticides were some of the causes of chemical injuries. Specific causes included iron and iron materials 74 (3.45%), grass, bamboo, and maize leaf 53 (2.50%), arc welding 45 (2.10%), and cement 19 (0.90%).

On evaluation of visual acuity at presentation and during their last follow-up, most of the patients had mild or no vision impairment. A total of 63 (2.94%) patients were classified as blind (<3/60) according to WHO criteria at presentation and 48 (2.23%) patients remained blind according to WHO criteria^6^ at their last follow-up.

## DISCUSSION

The prevalence of ophthalmic trauma varies according to geographic location, socio economic status, age groups, occupation and cultural practices.^1^ The importance of ocular trauma is not just how big the problem is, but the unique opportunity it brings to preventing it since 90 % of ocular trauma can be prevented by simple measures.^7^ The prevalence of ocular trauma in our study is 32.83% which is relatively high compared to 0.70% in Bhaktapur Eye Study, 2-10% in Andra Pradesh and Tamil Naidu, 9.40% in KLES hospital and 3.93% in North India.^8^ The first two were population-based studies and the last two were done in general hospital. Since our study was done in emergency department of Eye hospital, hence the higher prevalence rate.

Thylefors has estimated over half a million blind by eye injuries and up to 5% blind in developing world due to trauma.^9^ Due to the geographical, cultural and socioeconomic diversities in different parts of Nepal, the pattern of livelihood differs significantly, although 66% of total households still rely on agriculture for their living. In the Nepal Blindness survey done in 1981, trauma ranked fifth in the avoidable cause of blindness and it was the second leading cause of monocular blindness with an estimated 0.86% of ocular trauma in the country. Agriculture was mostly implicated with paddy husks, seeds, twigs, wood accounted for most cases.^10^ In our study agriculture and its products were cause of 223 (10.40%) cases of ocular trauma which may be an understatement since many causes of corneal and conjunctival foreign body were unknown. Hetauda, a metropolitan city, is a hub of cement factories, welding and iron industries and manufacturing products. In their cross-sectional interview based prospective study, Ben et al reported 38.30% of the workers experienced some form of ocular injury and 68.3% never wore safety gear at work.^11^

In our study 148 (6. 91%) of total ocular injury cases were chemical injuries which is lower than 7.7-22.10% mentioned in literature.^12^ Alkaline injuries are common than acidic due to their extensive use in industrial and domestic use and lime (chuna) was the most common cause of chemical injury followed by toilet cleaner in a study of ocular chemical injuries in the pediatric age group in India.^12^ In our study unidentified chemical, cement, superglue, paint were some causes of chemical injury. In our study, 1618 (75%) of ocular trauma patients were males, supporting the findings of other studies.^11-18^ Almost 90 % of our patients fall in the working age population which is quite alarming from socioeconomic point of view. The pattern of ocular trauma is our study is different from studies in the west; welding injury and workplace injury like cement factories and agriculture rank second and third in our studies, whereas welders comprised 5.1% and workers engaged in welding comprised 8.2% of all eye injuries in study done in USA.^17^ Occupational injury comprised 604 (28.20%) of ocular trauma cases as opposed to study in India and Queensland where non occupational injuries like sports and RTA were common causes.^2,13,14^ Road traffic accidents, physical assaults and sports constituted only 16 (0.75%) cases of ocular trauma cause in our study. In Taiwan, the most frequent cause of trauma was metallic object, while in our study, iron and metal caused only 98 (4.57%) of ocular trauma.^16^ In Singapore superficial foreign body, corneal abrasion and blunt trauma were the three most common cause of ocular injury resembling to our findings.^18^

The aim of our study was to find the prevalence of ocular trauma at the emergency department of HCEH and identify vulnerable circumstances and population for effective intervention. Pizarello et al mentioned that most of the trauma related ocular blindness can be prevented.^7^ Studies have shown reduction in ocular trauma with alcohol management plans and mandatory eye protection in sports.^3,4^ Prevention strategies and the introduction of eye protection have found to be effective in reducing the incidence of ocular trauma.^2^ Occupational trauma related to welding, industries and agriculture are common and can be targets for ocular injury prevention. The success stories of the west can be replicated with proper advocacy and planning.

The study had the inherent limitation of retrospective study with one year data, threfore the findings are applicable to this specific population only. We recommend further prospective studies with samples representing geographical variations. Moreover, finding out the association with the various causes would also add up valuable information for the prevention and management of occular trauma.

## CONCLUSIONS

Our emergency department ocular trauma prevalence was higher in comparison to other studies. Occupational trauma is a common circumstance of ocular trauma.

## References

[1] Ng SMS, Low R, Hoskin AK, Rousselot A, Gunasekeran DV, Natarajan S (2022). The application of clinical registries in ophthalmic trauma-the International Globe and Adnexal Trauma Epidemiology Study (IGATES).. Graefes Arch Clin Exp Ophthalmol..

[2] Hoskin AK, Watson SL (2020). Ocular trauma and prevention measures.. Clin Exp Ophthalmol..

[3] Lincoln AE, Caswell SV, Almquist JL, Dunn RE, Clough MV, Dick RW (2012). Effectiveness of the women's lacrosse protective eyewear mandate in the reduction of eye injuries.. Am J Sports Med..

[4] Dorman A, O'Hagan S, Gole G (2020). Epidemiology of severe ocular trauma following the implementation of alcohol restrictions in Far North Queensland.. Clin Exp Ophthalmol..

[5] Dandona L, Dandona R (2006). Revision of visual impairment definitions in the international statistical classification of diseases.. BMC Med..

[6] Gurung HB, Kalpana S, Sunil T Ocular Emergency Study (OES):True Ocular Emergency and Non-urgent Cases at Emergency Department at Hetauda Community Eye Hospital.. East African Scholars J Med Sci..

[7] Pizzarello LD (1998). Ocular trauma: time for action.. Ophthalmic Epidemiology [Internet]..

[8] Upadhyay MP, Karmacharya PC, Koirala S, Shah DN, Shakya S, Shrestha JK (2001). The Bhaktapur eye study: ocular trauma and antibiotic prophylaxis for the prevention of corneal ulceration in Nepal.. Br J Ophthalmol..

[9] Thylefors B (1992). Epidemiological patterns of ocular trauma.. Australian and New Zealand Journal of Ophthalmology [Internet]..

[10] Brilliant LB, Pokhrel RP, Grasset NC, Lepkowski JM, Kolstad A, Hawks W (1985). Epidemiology of blindness in Nepal.. Bull World Health Organ..

[11] Limbu B, Moore G, Marvasti A, Poole M, Saiju R (2018). Work Related Ocular Injury: Nepal.. Nepalese Journal of Ophthalmology.

[12] Akgun Z, Selver OB (2023). Epidemiology and etiology of chemical ocular injury: A brief review.. World J Clin Cases..

[13] Maurya RP, Srivastav T, Singh VP, Mishra CP, Al-Mujaini A (2019). The epidemiology of ocular trauma in Northern India: A teaching hospital study.. Oman J Ophthalmol..

[14] Park J, Yang SC, Choi HY (2021). Epidemiology and Clinical Patterns of Ocular Trauma at a Level 1 Trauma Center in Korea.. J Korean Med Sci.

[15] Kwon J woo, Choi MY, Bae JM (2020). Incidence and seasonality of major ocular trauma: a nationwide population-based study.. Scientific Reports [Internet]..

[16] Chang YS, Teng YT, Huang YH, Liu ML, Hung JH, Hsu SM (2018). Major ocular trauma in Taiwan: 2002-2004 versus 2012-2014.. Sci Rep..

[17] Lombardi DA, Pannala R, Sorock GS, Wellman H, Courtney TK, Verma S (2005). Welding related occupational eye injuries: a narrative analysis.. Inj Prev..

[18] Voon LW, See J, Wong TY (2001). The epidemiology of ocular trauma in Singapore: Perspective from the emergency service of a large tertiary hospital.. Eye [Internet]..

